# *Oryza sativa* Brittle Culm 1-like 6 modulates β-glucan levels in the endosperm cell wall

**DOI:** 10.1371/journal.pone.0217212

**Published:** 2019-05-23

**Authors:** Keiko Midorikawa, Masaharu Kuroda, Haruyuki Yamashita, Tomoko Tamura, Keiko Abe, Tomiko Asakura

**Affiliations:** 1 Department of Applied Biological Chemistry, Graduate School of Agricultural and Life Sciences, The University of Tokyo, Tokyo, Japan; 2 Division of Crop Development, Central Region Agricultural Research Center, National Agriculture and Food Research Organization (NARO), Niigata, Japan; 3 Department of Nutritional Science and Food Safety, Faculty of Applied Bioscience, Tokyo University of Agriculture, Tokyo, Japan; 4 Kanagawa Institute of Industrial Science and Technology (KISTEC), Life Science & Environmental Research Center (LiSE), Kanagawa, Japan; Universidade de Lisboa Instituto Superior de Agronomia, PORTUGAL

## Abstract

The endosperm cell wall affects post-harvest grain quality by affecting the mechanical fragility and water absorption of the grain. Therefore, understanding the mechanism underlying endosperm cell wall synthesis is important for determining the growth and quality of cereals. However, the molecular machinery mediating endosperm cell wall biosynthesis is not well understood. In this study, we investigated the role of *Oryza sativa* Brittle Culm 1-like 6 (OsBC1L6), a member of the COBRA-like protein family, in cellulose synthesis in rice. *OsBC1L6* mRNA was expressed in ripening seeds during endosperm enlargement. When OsBC1L6-RFP was expressed in *Arabidopsis* cell cultures, this fusion protein was transported to the plasma membrane. To investigate the target molecules of OsBC1L6, we analyzed the binding interactions of OsBC1L6 with cellohexaose and the analogs using surface plasmon resonance, determining that cellohexaose bound to OsBC1L6. The β-glucan contents were significantly reduced in *OsBC1L6*-RNAi calli and *OsBC1L6*-deficient seeds from a *Tos* insertion mutant, compared to their wild-type counterparts. These findings suggest that OsBC1L6 modulates β-glucan synthesis during endosperm cell wall formation by interacting with cellulose moieties on the plasma membrane during seed ripening.

## Introduction

The endosperm cell wall functions in a variety of processes in cereals, including morphogenesis and ripening, cell and tissue protection, polysaccharide storage, and the selective permeation of enzymes and hydrolysates during germination [1–3]. From a food processing standpoint, the cell wall is important because it determines the fragility, quality, and water-absorbing properties of grains [4–6].

Additional nitrogen fertilization at heading time affects rice quality. To explore the mechanism underlying this effect, we previously examined the changes in gene expression and components in rice seeds induced by nitrogen fertilization at the heading stage. Comprehensive gene expression analysis of ripening seeds showed that genes related to cell wall synthesis were downregulated during this process, particularly cellulose synthase (CESA) genes, e.g. *OsCESA2*, *OsCESA5*, *OsCESA6*, and *OsCESA8*. As a result, the levels of β-glucan (estimated based on Calcofluor White fluorescence) were reduced in the endosperm of mature seeds [7]. Changes in cell wall components in response to nitrogen fertilization reduce the mechanical strength of cell walls in the plant body, thereby weakening disease and lodging resistance [8–10]. Similarly, the mechanical strength of rice seeds decreases in response to nitrogen fertilization, but little is known about the molecular biology of cell wall formation in seeds, particularly the endosperm, which occupies most of the seed.

In addition to various *CESA* genes, *Brittle Culm 1-like 6* (*OsBC1L6*) is downregulated in rice in response to additional nitrogen fertilization [7]. OsBC1L6 is a member of the COBRA-like family, whose members are involved in cellulose synthesis [11,12]. The first *COBRA*-like gene identified in plants was found in *Arabidopsis thaliana* [13]. The *Arabidopsis COBRA*-deficient mutant *cob4* shows severe growth defects accompanied by a disruption of microfibril orientation [14]. Many *COBRA*-like genes have since been identified in other plant species. For example, *Brittle Culm 1* (*OsBC1*) is a *COBRA*-like gene controlling the cellulose content and mechanical strength of rice culms [15]. Homology searches revealed 10 additional *OsBC1-like* (*OsBC1L1-10*) genes in the rice genome [16]. Phenotypic analysis was previously performed on T-DNA or *Tos17* insertion mutants of seven *OsBC1-like* genes, *OsBC1*, *OsBC1L2*, *OsBC1L3*, *OsBC1L4*, *OsBC1L5*, *OsBC1L6*, and *OsBC1L9*. Among these, the *Osbc1l4* mutant shows a severe dwarf phenotype, with few tillers and reduced cellulose contents in its cell walls [17]. Moreover, pollen tube elongation is inhibited in *Osbc1l5* [16]. By contrast, no significant phenotypic variation was observed in the insertion lines, except for *Osbc1l4* and *Osbc1l5* [16]. However, the endosperm cell walls in these mutants were not examined in detail, and the roles of COBRA-like proteins in endosperm cell wall synthesis have not yet been reported.

Among members of the *OsBC1L* family, *OsBC1L6* is expressed in seeds at the ripening stage, especially in the endosperm, as revealed by examining the Genevestigator database (https://genevestigator.com/gv/) (S1 Fig) and as reported in a previous study [16]. In this study, we demonstrate that *OsBC1L6* is a novel cell wall-related gene in the *OsBC1L* family that contributes to β-glucan synthesis in rice.

## Materials and methods

### Plant materials

**Fig 1:**
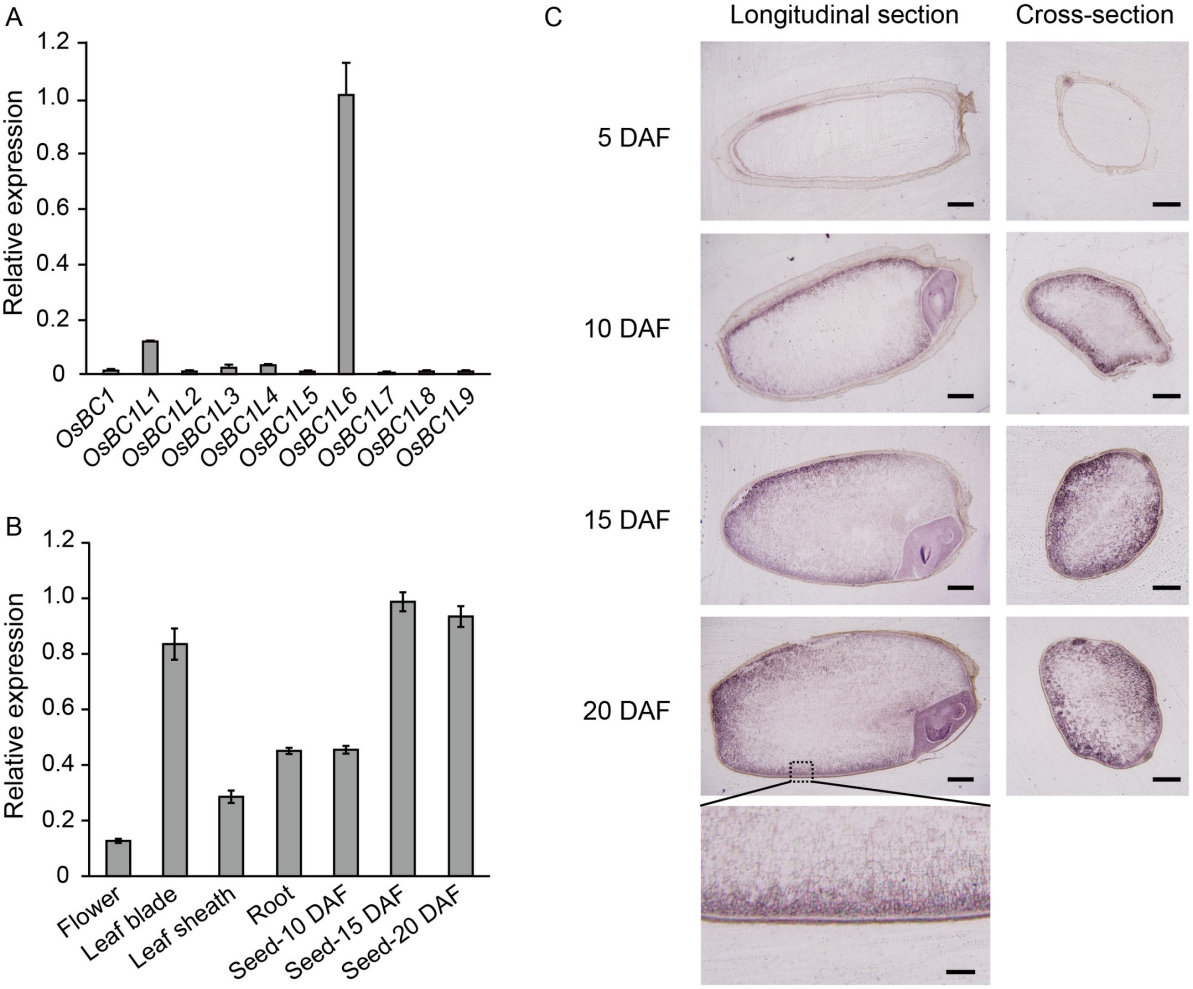
Expression analysis of *OsBC1L6*. (A) Comparison of the expression levels of *OsBC1* gene family members in 15-DAF seeds. (B) *OsBC1L6* expression levels in each tissue. (A, B) Values were normalized to the expression level of *RUBQ*. The primers are listed in S1 Table. Data represent the mean ± SD for three independent samples. (C) Expression analysis of *OsBC1L6* by *in situ* hybridization. Time course of *OsBC1L6* expression in ripening seeds. The probe was targeted to a 390-bp region of *OsBC1L6* including the coding region (206 bp) and 3′-UTR (184 bp). Scale bars = 0.5 mm. The lower panel is an enlarged image of the region of the longitudinal section of a 20-DAF seed indicated by a dotted line. Scale bar = 0.1 mm.

*Oryza sativa* L. cv. Nipponbare was used in all experiments. The cultivation in the plant incubator and sampling the ripening seeds were performed as previously described [7]. These plants were cultivated with short-day conditions under a 12-h-light (28°C)/12-h-dark (22°C) cycle. To obtain the samples for RT-PCR analysis shown in Fig 1B, plants were grown in a greenhouse under short-day conditions with a 12-h-light (30°C)/12-h-dark (25°C) cycle. All tillers were removed during cultivation so that each plant had only one panicle.

### RT-PCR and gene expression analysis

Total RNA was isolated from the samples using TRIzol reagent (Thermo Fisher Scientific, Waltham, MA, USA) with additional DNase treatment (Qiagen, Hilden, Germany). cDNA was synthesized from RNA template (3 μg) using SuperScript IV Reverse Transcriptase (Thermo Fisher Scientific) according to the manufacturer’s instructions. Floral tissues were collected from 82-day-old seedlings, leaf blades and sheaths were collected from 81- to 97-day-old seedlings, and roots were collected from 105-day-old seedlings. All samples were flash-frozen in liquid nitrogen and stored at -80°C until use. PowerUp SYBR Green Master Mix (Thermo Fisher Scientific) was used on an ABI 7500 real-time PCR system (Thermo Fisher Scientific). The thermal cycling program was performed using the following parameters: denaturation at 95°C for 2 min, followed by 40 amplification cycles (95°C for 15 sec, 60°C for 1 min). Melting curves were performed after 40 cycles to confirm the specificity of the reactions. The 2^-ΔΔCT^ method was used to calculate the relative expression of *OsBC1L6* following normalization to the housekeeping gene *Ubiquitin* (*RUBQ*, Os02g0161900). At least two biological replicates and three technical replicates were performed for data analysis. The primer sequences are provided in S1 Table.

### *In situ* hybridization

*In situ* hybridization was performed as previously described with minor modifications [18,19]. Seed samples (5, 10, 15, and 20 days after flowering, [DAF]) were soaked in 50 mM phosphate buffer (pH 7.2) containing 4% (w/v) paraformaldehyde and 0.25% (v/v) glutaraldehyde, degassed for 30 min with an aspirator (MDA-015, ULVAC, Tokyo, Japan), and fixed overnight at 4°C. After fixation, the fixative was replaced with 3% (w/v) carboxymethyl cellulose. Frozen sections (5 μm thick) were prepared as described [7].

To construct the *OsBC1L6* probe, a 390-bp fragment of the 3′ region of its cDNA, including the 206-bp coding region and 184-bp 3′-UTR specific to *OsBC1L6*, was subcloned into the pBlueScriptII SK+ vector (Agilent Technologies, Santa Clara, CA, USA) as a template for *in vitro* transcription. The specificity of the probe for *OsBC1L6* was confirmed through BLAST searches (http://www.ncbi.nlm.nih.gov/). Besides *OsBC1L6*, the probe sequence shares the highest homology (only 29%) with *OsBC1L4*, indicating that the probe sequence is based on a highly specific region among members of the *OsBC1L* family. The digoxigenin (DIG)-labeled antisense RNA probe was generated with T3 polymerase according to the instructions for the DIG RNA Labeling Kit (Roche, Basel, Switzerland). The primers used are listed in S2 Table. Hybridization was performed as described previously [19]. No specific signal was detected in the equivalent section probed with the *OsBC1L6* sense probe (S2 Fig). The prepared slides were observed under a light microscope (BX60, Olympus, Tokyo, Japan).

### *In silico* analysis of the amino acid sequence of OsBC1L6

The signal peptide and GPI anchor were predicted with SignalP (www.cbs.dtu.dk/services/SignalP) [20] and big-PI predictor (http://mendel.imp.ac.at/gpi/gpi_server.html) [21–24]. Phyre2 [25] (http://www.sbg.bio.ic.ac.uk/phyre2) was used to predict the secondary structure and CBM region of OsBC1L6. The transmembrane region was predicted with TMHMM (http://www.cbs.dtu.dk/services/TMHMM/) [26,27].

### Subcellular localization

To verify the subcellular localization of OsBC1L6, various plasmids were transiently expressed in “Deep” cells as described previously [28]. To avoid cleavage of OsBC1L6 during protein modification, RFP was inserted into OsBC1L6 at a position 29 amino acids away from the N terminus. For the template, the full-length cDNA clone of *OsBC1L6* obtained from the Genetic Resources Center, NARO (Tsukuba, Japan), was used. First, In-Fusion cloning was conducted to insert an 84-bp signal sequence between the 35S promoter and RFP. To generate the linear vector, inverse PCR was performed from the signal sequence insertion site, after which In-Fusion cloning was performed according to the manufacturer’s protocol (In-Fusion HD Cloning Kit, Clontech-Takara Bio USA, CA, USA). Subsequently, the remaining *OsBC1L6* sequence, except for the signal sequence, was amplified by PCR. Five amino acid residues, GGSGG, were added as a linker to the N terminus of *OsBC1L6*. The PCR fragments were inserted into binary vector pTU10 in the correct orientation after digestion with *Bam*HI. The primers used to generate the OsBC1L6-RFP vector are listed in S2 Table. The transformed cells were observed under a confocal laser scanning microscope (FV10i, Olympus).

### Protein expression and purification

The CBM region of OsBC1L6 was identified by homology analysis based on the secondary structure of the protein using the Phyre2 database (http://www.sbg.bio.ic.ac.uk/phyre2) for protein structure prediction [25]. DNA fragments corresponding to the CBM of OsBC1L6 ^31^Pro–^191^Thr were inserted in-frame into pEU-GST. To express and purify CBM-GST, the expression vector constructs were transcribed and translated using a WEPRO7240G Wheat Germ Cell-free Expression Kit (CellFree Sciences, Matsuyama, Japan) according to the manufacturer’s instructions. The purification was performed at 4°C as follows. The translation mixture was filtered through a 0.22-μm pore filter. The supernatant was applied to an open column packed with 400 μl of Glutathione Sepharose 4B (GE Healthcare, Little Chalfont, UK) equilibrated with buffer A (20 mM HEPES, pH 7.4, 150 mM NaCl, 1 mM DTT, and 0.1% Triton X-100). The column was washed with 10 column volumes of buffer A, and the proteins were eluted with 20 mM glutathione in buffer B (100 mM HEPES, pH 8.0, 150 mM NaCl, and 0.1% Triton X-100). The eluted solution was dialyzed overnight against a buffer consisting of 10 mM HEPES (pH 7.4) and 150 mM NaCl (GE Healthcare). The purity of the protein was verified by SDS-PAGE. The purified proteins were stored at 4°C until use.

### Surface plasmon resonance

Surface plasmon resonance (SPR) experiments were performed on a Biacore T200 system (GE Healthcare) according to the manufacturer’s instructions. Glutathione *S*-transferase (GST) antibody was covalently immobilized on a CM5 sensor chip at a density of ~12,000 resonance units (RUs) using a GST Capture Kit (GE Healthcare). Recombinant GST-fused CBM (CBM-GST) in BC1L6 was captured on the sensor chip by injection at a flow rate of 10 μL/min for 600 s with a stabilization period of 30 s. Anti-GST antibody and CBM-GST were diluted in HBS-EP running buffer (0.01 M HEPES, pH 7.4, 0.15 M NaCl, and 0.005% v/v Surfactant P20, GE Healthcare) to obtain a final concentration of 10 to 30 μg/mL. Affinity analyses of the interaction between CBM-GST and oligosaccharides were performed using a 30-μL/min flow rate at 25°C using HBS-EP buffer as the running buffer. CBM-GST was immobilized onto the Fc2 channel of a CM5 chip as the ligand; Fc1 was treated in the same manner but without CBM-GST as the control channel. Binding responses were obtained by subtracting Fc1 from Fc2 for each series of oligosaccharide concentrations (50, 100, 300, 500, 800, and 1000 μM). The oligosaccharides xylohexaose (cat. no. O-XHE), cellohexaose (cat. no. O-CHE), and 3^3^-α-L-arabinofuranosyl-xylotetraose (cat. no. O-XA3XX) were obtained from Megazyme (Bray, Ireland). The sensor surface was regenerated using a 10 mM glycine-HCl solution (pH 2.2) after each binding cycle. The data were analyzed using Microcal Origin version 1.0 (Microcal Software Limited) to obtain kinetic parameters.

### RNAi vector construction

Full-length *OsBC1L6* cDNA was obtained from the Genetic Resource Center, NARO (Tsukuba, Japan). A partial fragment of *OsBC1L6* corresponding to the 3′-UTR (S2 Table) was amplified for RNAi vector construction using pZH2Bik [29].

### *Agrobacterium*-mediated transformation of rice

*Agrobacterium*-mediated transformation of rice (*O*. *sativa* L. cv. Nipponbare) was performed following the protocol of Toki et al. [30], except that the *Agrobacterium* was disinfected after co-cultivation using 12.5 mg L^-1^ meropenem (Wako Pure Chemical Industries, Osaka, Japan) instead of carbenicillin. The co-cultured calli were transferred to N6D medium containing 50 mg L^-1^ hygromycin with 12.5 mg L^-1^ meropenem.

### Protoplast preparation and cell wall regeneration

For protoplast isolation, calli were harvested 4 days after subculture. The calli were suspended in a filter-sterilized enzyme solution containing 1% Cellulase Y-C (MP Biomedicals, Santa Ana, CA, USA), 0.5% Macerozyme R10 (Research Products International, Mount Prospect, IL, USA), 0.4 M mannitol, 10 mM CaCl_2_, and 5 mM MES (pH 5.6). After 3 h of incubation at 30°C with shaking at 90 rpm, the protoplasts were collected through a nylon mesh (125 μm pore), and the filtered solution was centrifuged at 800 rpm for 15 min. The protoplasts were washed in 10 mL of protoplast suspension medium (117 mM KCl, 82 mM MgCl_2_, and 80 mM CaCl_2_), and the mixtures were centrifuged at 800 rpm for 8 minutes. After the protoplasts were washed, they were cultured in sealed Petri dishes in protoplast suspension medium at a density of 1 x 10^5^ cells mL^-1^ in complete darkness at 30°C. Cell wall regeneration was evaluated by monitoring the fluorescence of 0.05% Calcofluor White under a microscope (BX51, Olympus).

### Identification of the *Tos17* insertion sites in *OsBC1L6*

The *Tos17* insertion line *Osbc1l6* (NF7793) was obtained from the *Tos17* Insertion Mutant Database (https://tos.nias.affrc.go.jp/). After each seed germinated, genotyping of the *Tos17* insertion was performed using *OsBC1L6*-specific primers NF7793-F and NF7793-R, along with *Tos17*-specific primer *Tos17*-tail 6 (S2 Table). In addition to selecting homozygous and heterozygous individuals, segregated wild-type individuals (segWT) in which the *Tos17*-inserted BC1L6 allele was lost through segregation were selected as the control. Seeds from the next generation were harvested from each individual independently, and homozygous individuals from heterozygous lines were identified through genotyping.

### β-glucan staining

β-Glucan staining was performed as described previously [7]. To compare the β-glucan contents of the *Osbc1l6* mutants, revertants with *Tos17* deleted from *OsBC1L6* were used as the wild type (segWT); an individual judged to be a homozygous *OsBC1L6* knockout line (strains 7–5 and 11–4) was used as the mutant strain. All images were processed using ImageJ software (https://imagej.nih.gov/ij/). The background was subtracted from the assignment area to calculate the fluorescence intensity per unit area.

## Results

### Expression analysis of *OsBC1L6*

In ripening seeds at 15 days after flowering (DAF), *OsBC1L6* showed the highest expression level among *OsBC1L* family members (Fig 1A). *OsBC1L6* was mainly expressed in leaf blades and seeds at 15 and 20 DAF (Fig 1B). To reveal the sites of *OsBC1L6* expression in ripening seeds, we performed *in situ* hybridization using frozen sections prepared from seeds at 5, 10, 15, and 20 DAF. *OsBC1L6* was initially expressed in seeds at 5 DAF, and its expression level increased during seed growth (Fig 1C). Notably, *OsBC1L6* was not expressed in the aleurone layer but was expressed in the region surrounding the starchy endosperm in the dorsal side of the seed. These results indicate that *OsBC1L6* is expressed in the starchy endosperm throughout development.

### Subcellular localization of OsBC1L6

**Fig 2:**
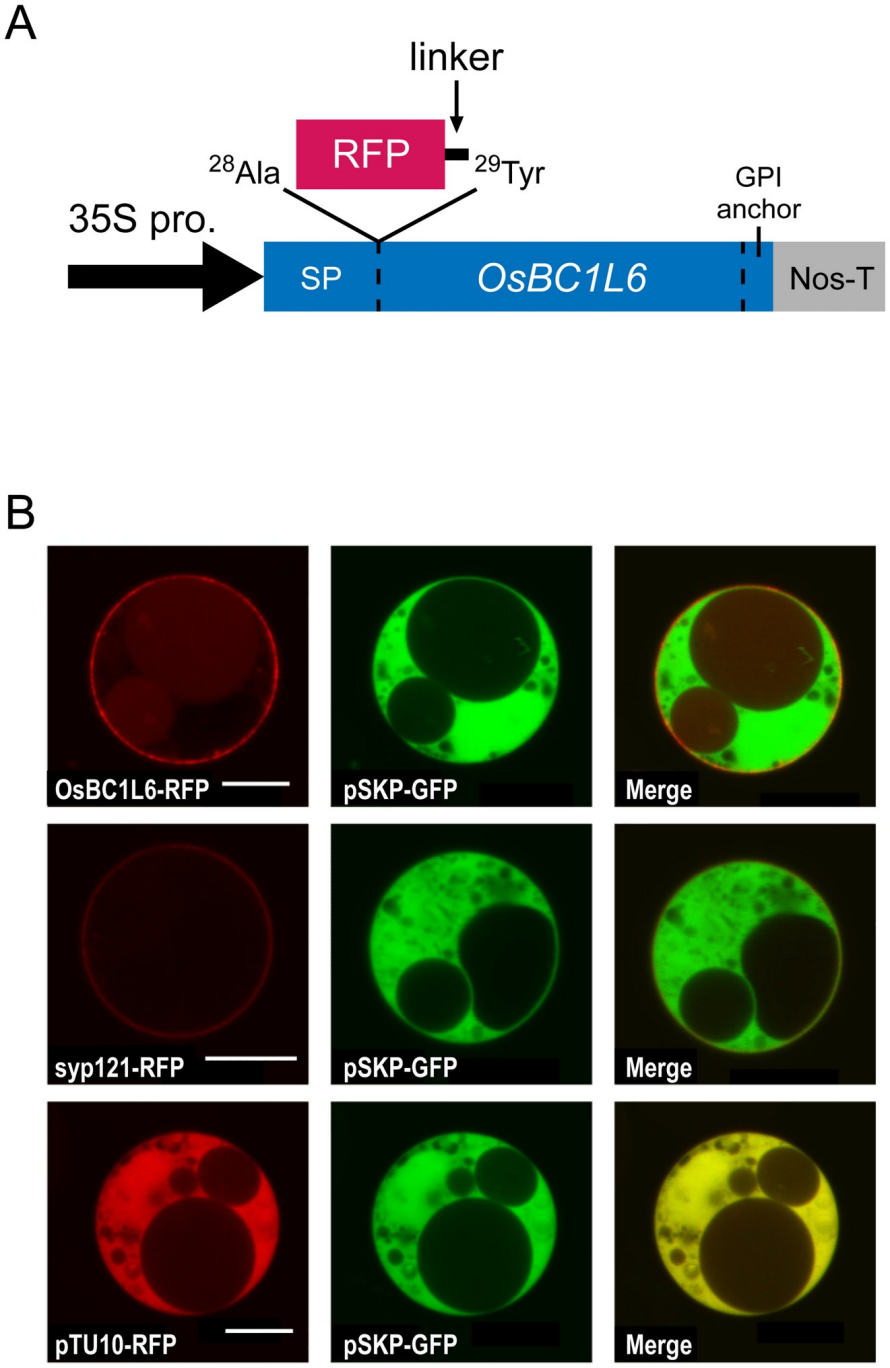
Subcellular localization of the OsBC1L6-RFP fusion protein in a transient expression assay. (A) Construct used for protoplast transformation. According to the predicted structure of OsBC1L6, the signal sequence ends at the 28th alanine (^28^Ala), and the mature region begins at the 29th tyrosine (^29^Tyr). RFP and linker amino acids (GGSGG) were inserted between ^28^Ala and ^29^Tyr. The fusion protein was expressed under the control of the 35S promoter from cauliflower mosaic virus (35S-Pro) and the nopaline synthase terminator from *Rhizobium radiobacter* (Nos-T). (B) OsBC1L6-RFP was transiently expressed in protoplasts isolated from cultured *Arabidopsis* root cells. As a control, the cells were cotransfected with pSKP-GFP expressing only GFP. Syp121-RFP was used as a plasma membrane marker and pTU10-RFP was used as a cytoplasmic and nuclear marker. After overnight culture in the dark, fluorescent signals were observed by confocal microscopy. Scale bar = 10 μm.

*In silico* analysis of the amino acid sequence of OsBC1L6 suggested the presence of a putative signal peptide and glycosylphosphatidylinositol (GPI) anchor protein site (S3 Fig). GPI anchor proteins are usually transferred to the surface of the plasma membrane and are sometimes released into the extracellular space and cell wall [31–33]. We confirmed the intracellular localization of OsBC1L6 by transiently expressing RFP-fused OsBC1L6 protein in cell cultures derived from *Arabidopsis* roots. Both the signal peptide and GPI anchor domain might be cleaved from mature BC1L6 protein. Therefore, we attached RFP to the N terminus of mature OsBC1L6 (Fig 2A). As a control, we cotransfected protoplasts with pSKP-GFP expressing only GFP. Syp121-RFP was used as a plasma membrane marker [34] and pTU10-RFP as a cytoplasmic and nuclear marker. The GFP signals overlapped completely with free RFP signals and were localized to the cytoplasm and nucleus (Fig 2B). Signals from OsBC1L6 fused to RFP did not overlap with GFP signals, and their localization was consistent with that of syp121-RFP signals. These results indicate that OsBC1L6 is transported at least to the plasma membrane.

### Interaction of OsBC1L6 with oligosaccharides

**Fig 3:**
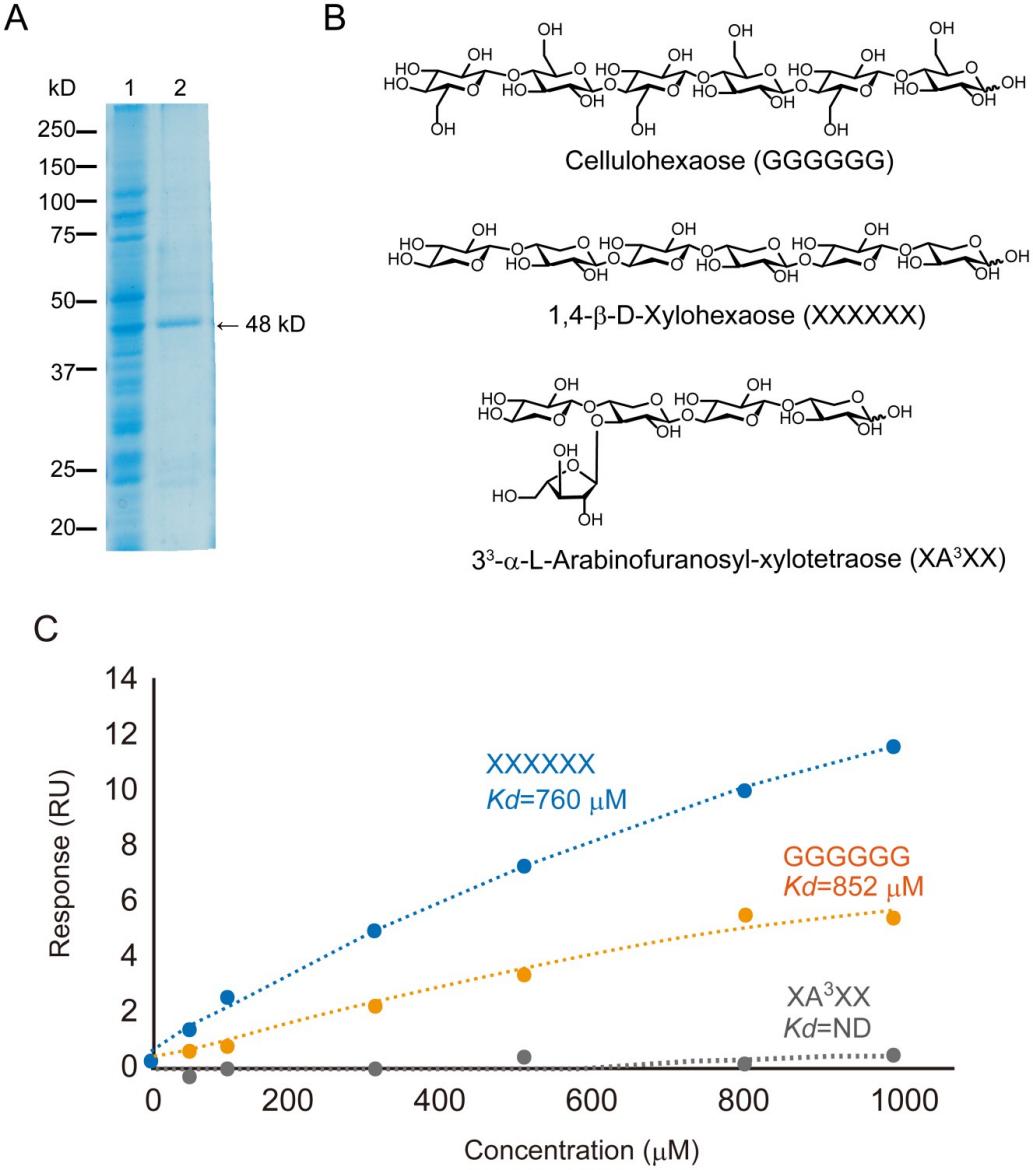
Analysis of OsBC1L6 binding to oligosaccharides by SPR. (A) SDS-PAGE of recombinant protein expression and purification. Lane 1, total extract from wheat germ cells; lane 2, purified CBM-GST recombinant protein in eluate. (B) Chemical structures of the oligosaccharides used as analytes. (C) Binding curves derived from SPR data for CBM-GST binding to oligosaccharides (50, 100, 300, 500, 800, and 1000 μM). *K*_d_ values are shown (ND = not determined).

OsBC1, a member of the OsBC1L family, is thought to modulate cellulose synthesis in cell walls [33]. OsBCL1 family members contain a CBM [35], which is thought to interact with a target carbohydrate chain. Since the endosperm cell wall in rice seeds is predominantly composed of approximately 23 to 30% cellulose, 40 to 50% hemicellulose, 10 to 27% pectin, and 1% lignin, and since arabinoxylan is the main component in hemicellulose [36–39], we examined the interaction between the main components of the endosperm cell wall (cellulose and arabinoxylan) and the CBM region of OsBC1L6 using SPR. To facilitate protein expression and purification of the recombinant protein, we expressed the CBM of OsBC1L6 as a fusion protein joined to the N terminus of glutathione *S*-transferase (CBM-GST) in a wheat germ cell-free expression system and subjected it to affinity purification. The affinity-purified protein was confirmed to form a single 48-kD band when analyzed by SDS-PAGE (Fig 3A).

CBM-GST was immobilized onto the sensor chip as a ligand and tested for interaction with oligosaccharides as analytes. Three oligosaccharides were examined in this experiment, xylohexaose (XXXXXX), cellohexaose (GGGGGG), and arabinoxylan oligosaccharide (XA^3^XX) (Fig 3B). Xylohexaose and cellohexaose are both linear hexasaccharides with β-1,4 linkage; arabinoxylan oligosaccharide is a branched pentasaccharide with the formula 3^3^-α-L-arabinofuranosyl-xylotetraose. A concentration-dependent interaction was detected with xylohexaose and cellohexaose, but not with arabinoxylan oligosaccharide (Fig 3C). The *K*_d_ value was 760 μM for xylohexaose and 852 μM for cellohexaose and was not determined for arabinoxylan oligosaccharide.

### Phenotypes of the *OsBC1L6*-RNAi line and knockout mutant

**Fig 4:**
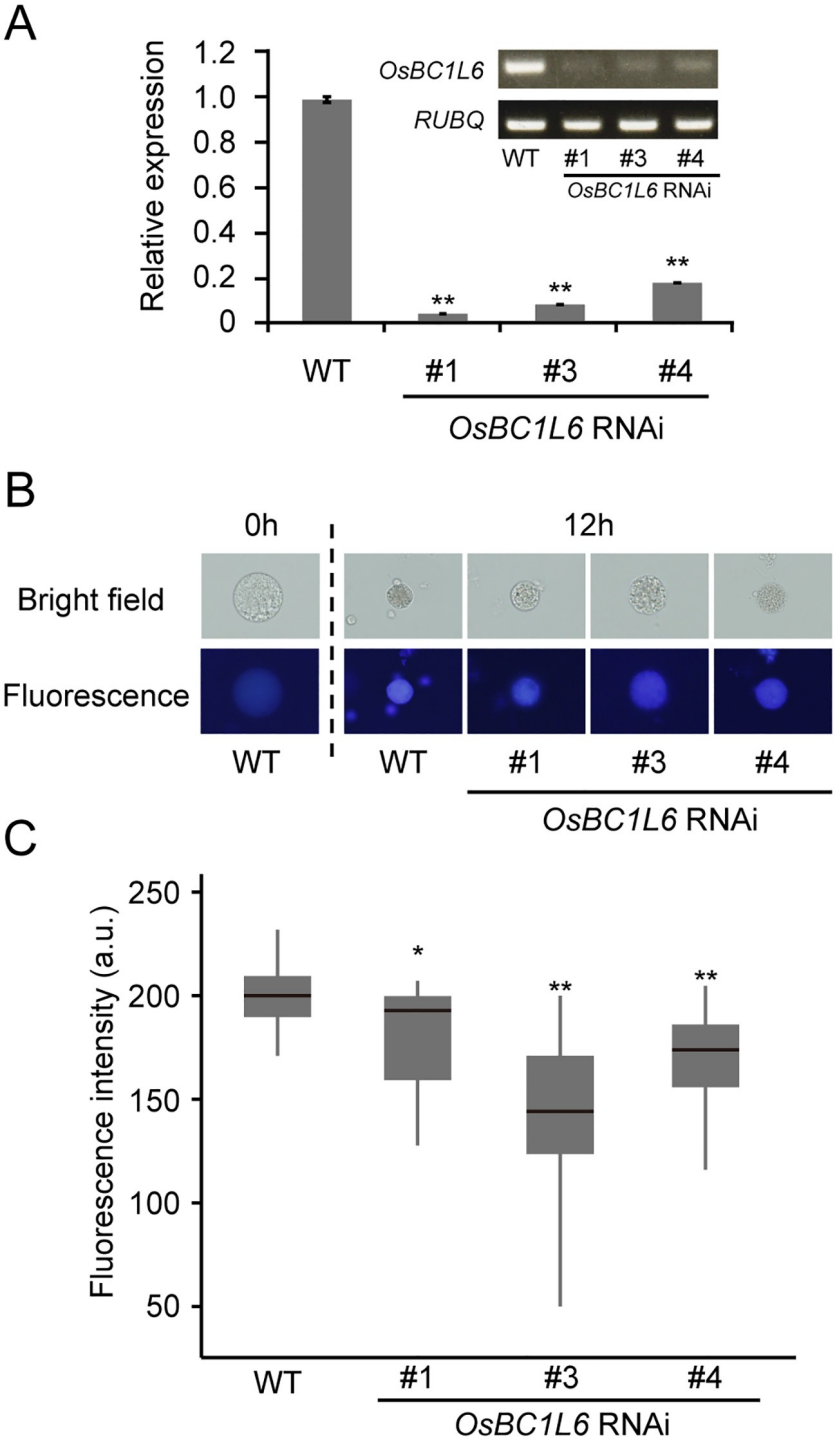
Cell wall regeneration in protoplasts isolated from *OsBC1L6*-RNAi lines. (A) The expression levels of *OsBC1L6* in *OsBC1L6*-RNAi calli were compared by semiquantitative RT-PCR (gel electrophoresis; upper right) and real-time PCR analysis (bar graph). Real-time PCR data were normalized to the expression levels of *RUBQ* and are shown relative to the wild type (WT). Data shown are mean ± SD for three biological replicates. (B) Protoplasts were stained with Calcofluor White, and samples from WT and *OsBC1L6*-RNAi (#1, #3, and #4) were observed in protoplasts after 12 h of cultivation. (C) Fluorescence intensity per area measured using ImageJ image analysis software; 20 individual calli from the WT and *OsBC1L6*-RNAi lines were analyzed. The fluorescence intensity values of WT and *OsBC1L6*-RNAi are represented in a box plot, with asterisks indicating significant differences, as determined by Wilcoxon rank sum test. a.u., arbitrary units. (*p < 0.05, **p < 0.01, n = 20).

To investigate the phenotype caused by the loss of *OsBC1L6* function, we generated RNAi calli with specifically suppressed expression of *OsBC1L6*. We analyzed the expression level of *OsBC1L6* in three *OsBC1L6*-RNAi lines (#1, #3, and #4) by RT-PCR (Fig 4A). In wild-type calli, the most strongly expressed *OsBC1L* genes were *OsBC1L1*, *OsBC1L4*, and *OsBC1L6* (S4A Fig). In the transgenic RNAi lines, *OsBC1L6* expression decreased to approximately 1/10th to 1/5th of wild-type levels (Fig 4A). These results confirm that the inhibition of mRNA expression was specific to *OsBC1L6* (S4B Fig). We produced protoplasts from *OsBC1L6*-RNAi calli and observed the progress of cell wall regeneration using Calcofluor White staining (Fig 4B). Following protoplast production, the protoplasts were subjected to shaking cultivation in standard culture medium. The fluorescence intensity was significantly lower in the RNAi lines compared to the wild type after 12 h of cultivation (Fig 4C), suggesting that cell wall synthesis was retarded in the *OsBC1L6*-RNAi lines.

**Fig 5:**
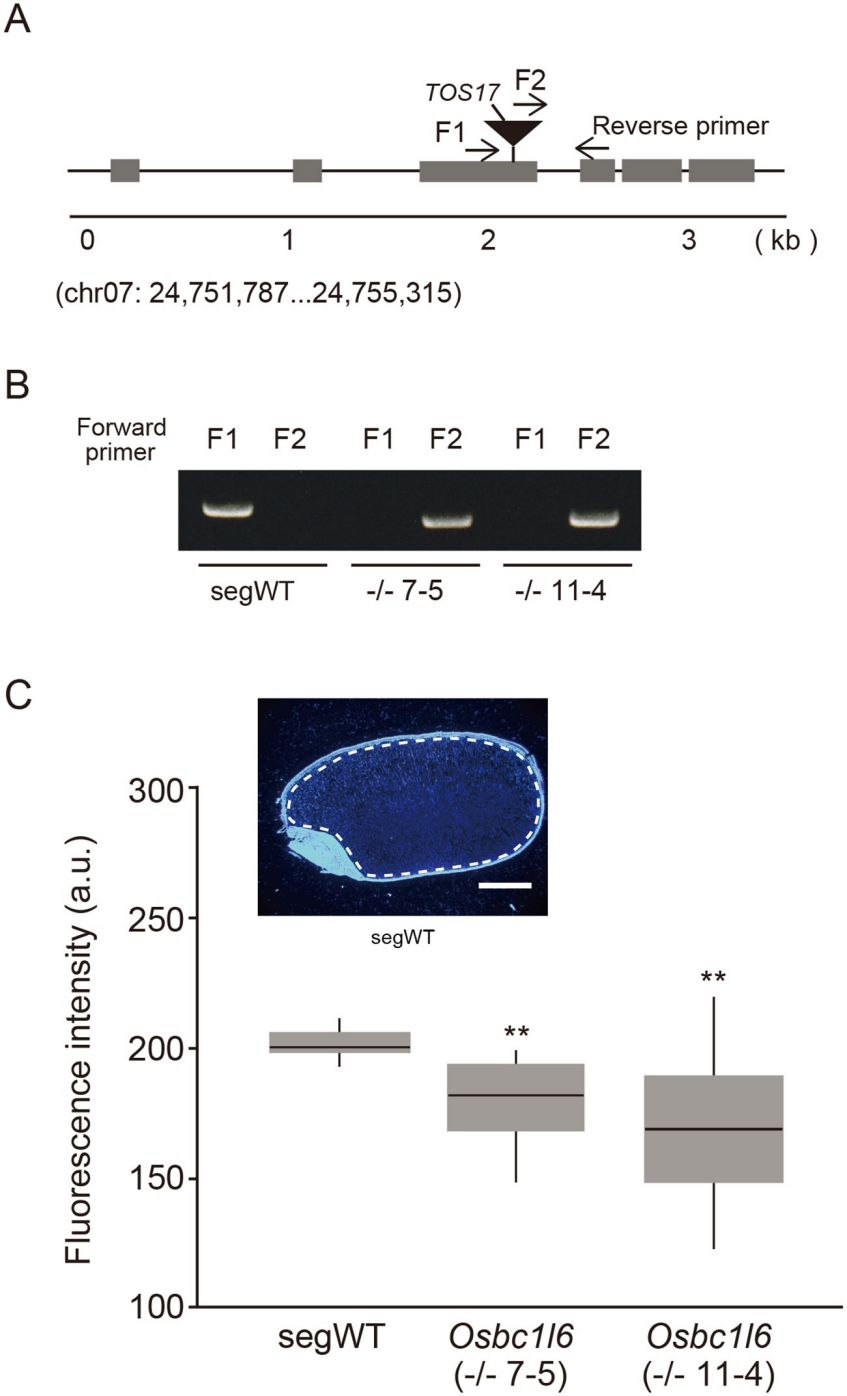
Phenotypic analysis of *Osbc1l6*. (A) Insertion site of the endogenous retrotransposon *Tos17* in the *Osbc1l6* mutant genome. The gray boxes represent the *OsBC1L6* exon. F1 (NF7793-F primer) and F2 (*Tos17*-tail6 primer) are forward primers. (B) Genotyping by PCR. In two lines (7–5 and 11–4), a PCR band appeared only when the primer combination F2 and Reverse was used, suggesting that they were *Tos17* insertion homozygous lines. In segWT plants, a PCR band appeared only when the primer combination F1 and Reverse was used. (C) Fluorescence intensity in endosperm analyzed using ImageJ. Sections were prepared from 20 randomly selected segWT and *Osbc1l6* grains. The sections were stained for β-glucan using Calcofluor White. The fluorescence intensity of the endosperm was calculated by adjusting the background intensity. (Upper left) Mature rice seed stained with Calcofluor White. Cross sections of grains from segWT are shown. The white dotted line indicates the area analyzed for fluorescence intensity. Scale bars = 1 mm. The fluorescence intensities of segWT and *Osbc1l6* are represented in a box plot, with asterisks indicating significant differences, as determined by Wilcoxon rank sum test. a.u., arbitrary units. (**p < 0.01).

We obtained *Osbc1l6*, a retrotransposon *Tos17* insertion mutant deficient in the *OsBC1L6* gene (Fig 5A), from the Rice *Tos17* Insertion Mutant Database [40,41]. Homozygous lines were identified based on PCR analysis of the genotype of each line (Fig 5B). RT-PCR analysis showed that the expression level of *OsBC1L6* was the most strongly reduced in *Osbc1L6* seeds among *OsBC1L* genes, but the expression levels of several other genes were affected as well (*OsBC1* and *OsBC1L1* were downregulated and *OsBC1L5* and *OsBC1L7* were upregulated) (S5 Fig). However, the appearance of *Osbc116* and its segWT plant were nearly identical; the stems and leaves did not appear fragile when we tried to break them off of the plants by hand. Similarly, the size of the *Osbc1l6* seeds did not differ from those of segWT (S6 Fig). To investigate the influence of *OsBC1L6* on the cell wall *in vivo*, we performed Calcofluor White staining using frozen sections prepared from mature seeds (Fig 5C, upper left). The fluorescence intensity was lower in the endosperm of *Osbc1l6* seeds compared to segWT, indicating that the deficiency of *OsBC1L6* in the mutant corresponded to the decrease in β-glucan levels in seeds (Fig 5C).

## Discussion

We previously reported that nitrogen fertilization at the heading stage reduces the expression of various genes for endosperm cell wall production [7]. In particular, reduced levels of β-glucan in the endosperm of fertilized seeds appear to be correlated with the reduced expression of genes involved in cellulose biosynthesis. Cellulose, a polysaccharide composed of (1, 4)-β-D-glucans, is a major component of the endosperm cell wall presumed to be involved in the mechanical strength and water permeability of polished rice [4–6]. Our results present a possibility that functional analysis of each fertilizer-responsive, cellulose-related gene discloses a relationship between cellulose and rice quality.

In this study, we focused on *OsBC1L6*, a member of the *COBRA*-like family. As expected by the database analysis (S1 Fig), RT-PCR analysis and *in situ* hybridization revealed that *OsBC1L6* was the most highly expressed *OsBC1L* family member in seeds (Fig 1B) and that it was specifically expressed in the endosperm (Fig 1C). This finding suggests that *OsBC1L6* mainly functions in the endosperm during seed development and is consistent with a previously performed comprehensive analysis of *OsBC1L* gene family members [16]. Although COBRA-like proteins are not cellulose synthases, many studies have revealed that the loss of these proteins leads to abnormalities in various traits related to cellulose synthesis [16,17,42–47], suggesting that COBRA-like proteins play important roles in plant growth through their effects on cellulose assembly, and they modulate cellulose crystallinity [33]. OsBC1L6 has two characteristic COBRA-like protein domains; a GPI-anchored domain and a CBM domain (S3 Fig). Both domains are important for the functions of these proteins: the GPI-anchored domain determines the intracellular localization of the protein to the plasma membrane, and the CBM domain interacts with polysaccharides.

In this study, subcellular localization analysis using OsBC1L6-RFP showed that OsBC1L6 localizes to the plasma membrane (Fig 2). The GPI anchor protein is sorted to the endoplasmic reticulum, and the GPI moiety is added in this compartment prior to transport to the plasma membrane [31,32]. We found that OsBC1L6-RFP was localized to the plasma membrane when we used cultured *Arabidopsis* cells to determine its subcellular localization. In an analysis of OsBC1 protein in rice using immunogold labelling, OsBC1 was found to be localized to the plasma membrane in cells with thin walls and to the cell wall in cells with thickened secondary cell walls [33]. Although *Arabidopsis* suspension cultured cell system cannot show the localization of molecules on the cell wall, OsBC1L6-RFP was actually found to be transported to at least plasma membrane. Considering the amino acid sequence similarity between OsBC1L6 and OsBC1, we insist that OsBC1L6 might be localized in to the cell wall as well as OsBC1. Further examination will be necessary to clarify the exact localization site of OsBC1L6.

Phylogenetic analysis classified the COBRA-like protein families in *Arabidopsis* and rice into two clusters (S7 Fig). OsBC1 and OsBC1L4 belong to the same subfamily as OsBC1L6. CBM of OsBC1 was estimated to be closest to CBM2 family members [33], which bind cellulose (CBM2a) and xylan (CBM2b) [48,49]. Both types of CBM2 protein contain aromatic amino acid residues that interact with ligands [50], and these residues are highly conserved in the CBM of COBRA-like proteins [33]. According to Liu et al. [33], the CBM of OsBC1 interacts with cellulose, and ^46^Tyr and ^72^Trp are critical residues for binding with cellulose and targeting to the cell wall. The CBM of OsBC1L6 also shares high similarity with OsBC1. Therefore, the targets of OsBC1L6 might be cellulose as well as OsBC1 [33].

To characterize the OsBC1L6 ligand, we analyzed the kinetic interactions between the CBM of OsBC1L6 and various oligosaccharides using SPR. The CBM of OsBC1L6 interacted with xylohexaose and cellohexaose, but not with arabinoxylan oligosaccharide (Fig 3C). Arabinoxylan has a backbone containing only xylose residues, but arabinoxylan in the rice endosperm cell wall has very high branching density [51], suggesting that the potential ligand of OsBC1L6 might be cellulose, not arabinoxylan. However, as interaction analysis by SPR is limited to the analysis of soluble molecules, the interaction with cellulose must be confirmed using other methods. The binding constant of OsBC1L6 CBM for cellohexaose was 852 μM (Fig 3C), indicating that it binds much more weakly to its targets compared to the binding of other CBM2-containing proteins such as xylanase and cellohexanase from bacteria [49,52].

COBRA-like proteins are thought to guide the molecular orientation of their target molecules [15,17,33], which might not require strong interactions with ligands. OsBC1L6 might coordinate cellulose crystallization or the orientation of cellulose fibers through moderate interactions with cellulose. Furthermore, *in situ* hybridization showed that *OsBC1L6* is expressed in the endosperm of developing seeds. Taken together, these results suggest that OsBC1L6 might help determine the orientation of cellulose filaments in the endosperm cell wall. Liu et al. [33] demonstrated that OsBC1 modulates cellulose assembly by interacting with cellulose and affecting microfibril crystallinity; the current results support this observation.

Finally, we confirmed the role of OsBC1L6 in cell wall formation by visualizing β-glucan with Calcofluor White staining. As shown in Fig 4C, the slower increase in fluorescence in protoplasts with suppressed *OsBC1L6* expression indicates that *OsBC1L6* contributes to cell wall formation in rice cells via β-glucan synthesis. In the mutant in which *OsBC1L6* was interrupted by the insertion of the *Tos17* transposon in the third exon (Fig 5A), the level of fluorescence in the endosperm area was lower than that of segWT (Fig 5C), suggesting that the deficiency of *OsBC1L6* expression reduces β-glucan levels in endosperm. Dai et al. [16] reported that the expression level of *OsBC1L6* was not modified in a T-DNA inserting line; however, the T-DNA in this line was inserted in the promoter region and therefore might not have affected *OsBC1L6* expression. By contrast, the expression level of *OsBC1L6* was severely reduced in our *Tos17*-insertion line (S5 Fig). These findings indicate that *OsBC1L6* contributes to β-glucan synthesis in the endosperm.

In addition to the biological function of OsBC1L6, our results suggest that the *Osbc1l6* mutant may be useful for rice breeding. Mutants in *COBRA*-like genes, such as *Osbc1*, *Osbc1l4*, and *Osbc1l5*, had a severe detrimental effect on crop productivity[15–17,33] and should therefore not be used for breeding. By contrast, *Osbc1l6* plants and seeds appear to have a normal phenotype (S6 Fig), except for reduced β-glucan content in the endosperm (Fig 5C). The disruption of cellulose orientation due to the lack of glucan synthase genes in grass mutants leads to seriously reduced physical strength and pathogen resistance [53,54]. From these reports, the effect of *OsBC1L6* deficiency is thought to be related to the physical properties of the cell walls. In conclusion, we found that OsBC1L6 is involved in the formation of β-glucan including cellulose in the endosperm cell wall of rice seed. If the reduction in β-glucan levels in *OsBC1L6* mutants alters cell wall strength, the resulting increase in water permeability would enhance starch gelatinization and improve the cooking quality of polished rice. Properties of *Osbc1l6* grains should be analyzed further to determine whether this mutation could contribute to development of novel rice cultivars.

## Supporting information

S1 Table PCR primers used for RT-qPCR. (PDF)

S2 Table PCR primer sequences. (PDF)

S1 Fig Expression patterns of *OsBC1L* family members in different rice tissues. The expression patterns of *OsBC1L* family members in different tissues obtained from the DNA microarray database Genevestigator (https://genevestigator.com/). *OsBC1L2*, *OsBC1L3*, and *OsBC1Lp1* are not listed because no probes for these genes are available in the GeneChip Rice Genome Array (Affymetrix).
(TIF)

S2 Fig *In situ* mRNA localization of *OsBC1L6* in developing seeds. Sections of 15-DAF seeds hybridized with an *OsBC1L6* antisense probe (left) and sense probe (right).
(TIF)

S3 Fig Primary amino acid sequence of OsBC1L6. The predicted amino acid sequence of OsBC1L6. The signal peptide sequence is underlined. The region expected to be a polysaccharide-binding domain is indicated by asterisks (CBM). The dotted line represents the CCVS Cys-rich domain, which is highly conserved across the *COBRA* gene family. The GPI anchor domain is boxed. After translation, this domain is expected to be cleaved at the ω site (arrowhead) of the N terminus of the GPI anchor domain, and the GPI anchor is added.
(TIF)

S4 Fig Relative expression of *OsBC1L* family members in WT and *OsBC1L6*-RNAi calli. Total RNA was extracted from WT and *OsBC1L6* RNAi calli (#1) and subjected to RT-PCR. (A) Expression levels of *OsBC1L* family members in WT calli. (B) In the *OsBC1L6*-RNAi line, the expression of *OsBC1L6* was specifically suppressed. Values were normalized to the expression level of *RUBQ* and are shown as the mean ± SD. Asterisks indicate significant differences, as determined by Student’s *t* test (**p < 0.01, n = 3). The primers used in this experiment are shown in S1 Table.
(TIF)

S5 Fig Relative expression of *OsBC1L* family members in segWT and *Osbc1l6*. Total RNA was extracted from 15-DAF seeds of WT and the *Tos17* insertion mutant (-/- 7–5) and subjected to RT-PCR. Values were normalized to the expression level of *RUBQ* and are shown as the mean ± SD. Asterisks indicate significant difference, as determined by Student’s *t* test (**p < 0.01, n = 3). The primers used in this experiment are shown in S1 Table.
(TIF)

S6 Fig Comparison of the phenotypes of segWT versus *Osbc1l6* seeds. (A) Grain length. (B) Grain width. (C) Grain weight. Data represent the mean ± SD (n = 50).
(TIF)

S7 Fig Phylogenetic analysis of OsBC1L6. Phylogenetic tree based on the amino acid sequences of the COBRA family proteins constructed using the CLC Sequence Viewer (https://www.qiagenbioinformatics.com/products/clc-sequence-viewer/). The tree was created using the neighbor joining method. The protein sequences of AtCOBRA (*Arabidopsis thaliana*) and OsBC1 (*Oryza sativa* cv. *japonica*) family members were obtained from the NCBI (https://www.ncbi.nlm.nih.gov). The signal peptide was predicted using SignalP version 4.1 (http://www.cbs.dtu.dk/services/SignalP/) [20], and the hydrophobic profile was generated using TMHMM version 2.0 (http://www.cbs.dtu.dk/services/TMHMM/). GPI modification was predicted using big-PI Predictor [21]. GenBank protein ID numbers are as follows: *Arabidopsis thaliana* AtCOB, AAK56072; AtCOBL1, AAF02128; AtCOBL2, BAB02996; AtCOBL3, AAG12670; AtCOBL4, CAC01762; AtCOBL5, BAB10644; AtCOBL6, AAB60732; AtCOBL7, CAA74765; AtCOBL8, BAB00585; AtCOBL9, BAB10345; AtCOBL10, BAB01166; AtCOBL11, CAB38841; rice (*Oryza sativa*) OsBC1, BAS84702; OsBC1L1, BAS83769; OsBC1L2, BAS84703; OsBC1L3, BAS86439; OsBC1L4, BAS93810; OsBC1L5, BAS99187; OsBC1L6, BAT02549; OsBC1L7, BAT02550; OsBC1L8, BAT03332; OsBC1L9, BAT11495; OsBC1Lp1, BAS90288.
(TIF)

## Abbreviations

BC1L: Brittle Culm 1-like
CBM: carbohydrate-binding module
DAF: days after flowering
DIG: digoxigenin
GPI: glycosylphosphatidylinositol
GST: glutathione *S*-transferase
segWT: segregated to wild type
SPR: surface plasmon resonance
