# Supplementary material for: Oryza sativa Brittle Culm 1-like 6 modulates β-glucan levels in the endosperm cell wall
Source: PLoS One. 2019 May 23;14(5):e0217212. doi: 10.1371/journal.pone.0217212 (PMC6532911; doi:10.1371/journal.pone.0217212)
Supplement: S1 Table — (PDF) [file pone.0217212.s001.pdf]

Table S1. Sequence of PCR primer for Real-time qPCR.

Gene name	RAP ID	Forward primer	Reverse primer
OsBC1	Os03g0416200	5'-TGGTGGCATGATCAGAAAAA-3'	5'-AGACCAAAATGCCCCTACCT-3'
OsBC1L1	Os03g0301200	5'-CGTTGCTCCGATTCTTTCAT-3'	5'-AATTGCTGCCAGATTCTGCT-3'
OsBC1L2	Os03g0416250	5'-AGGTCCATTCACCACAGACG-3'	5'-TGGCTGCCAGAATAAGAAGC-3'
OsBC1L3	Os03g0754500	5'-CACGCTGAGGAAAGCTGATAC-3'	5'-AAGTTCACCAAAGTGCTGTGC-3'
OsBC1L4	Os05g0386800	5'-AGTTAGGATGTTGAGCGACC-3'	5'-ATCCACTGGCATGTGAAACTC-3'
OsBC1L5	Os06g0685100	5'-GGTTGTGGACTCCTTGTGCT-3'	5'-AGCACCCTCAAACGCTAAAA-3'
OsBC1L6	Os07g0604300	5'-GGAGATAATCTGGGCGATGA-3'	5'-CAGGTCCACGATCTCAGGAT-3'
OsBC1L7	Os07g0604400	5'-CTAAACACGGCCCTTGATGC-3'	5'-AGCCCATAGAACATCGCCGTA-3'
OsBC1L8	Os07g0690900	5'-TTGTCGCTGCTGTAAAAACG-3'	5'-ATCACAACCCTAGCCCTCCT-3'
OsBC1L9	Os10g0497700	5'-ATTTCACCGAATGGAACCTG-3'	5'-GCCGACTTGGATTGACTTGT-3'
RUBQ	Os02g0161900	5'-TCCGTGGTGGTCAGTAATCA-3'	5'-ACGGACTCGATGGTCCATTA-3'
