# Supplementary material for: Oryza sativa Brittle Culm 1-like 6 modulates β-glucan levels in the endosperm cell wall
Source: PLoS One. 2019 May 23;14(5):e0217212. doi: 10.1371/journal.pone.0217212 (PMC6532911; doi:10.1371/journal.pone.0217212)
Supplement: S2 Table — (PDF) [file pone.0217212.s002.pdf]

Table S2. Sequence of PCR primers.

**Primer types**	**Primer name**	**Sequence**
in situ hybridization	ISH_*BC1L6*-F	5'-ATGAATTCTGTTCAAGAAGGACCCCAAG-3'
	ISH_*BC1L6*-R	5'-ATGAGCTCTGTGTTTGCATCTTATCTTGTCTG-3'
	T7 promoter primer	5'-GTAATACGACTCACTATAGGGC-3'
	T3 promoter primer	5'-CCCTTTAGTGAGGGTTAATT-3'
OsBC1L6-RFP vector construction	Inverse PCR_F	5'-TGGAGAGGGGGATCCATGGCGCTCCTGCTGCTG-3'
	Inverse PCR_R	5'-GTCCTCGGAGGAGGCTGCCTCTGACGACGGAAT-3'
	Insert Primer_F	5'-GCCTCCTCCGAGGACGTCAT-3'
	Insert Primer_R	5'-GGATCCCCCTCTCCAAATGAA-3'
	BamHI-linker-*BC1L6*_F	5'-AAGGATCCGGTGGATCTGGTGGATACGATCCGCTTGATCCAAATG-3'
	BamHI-*BC1L6*_R	5'-AAGGATCCCTATGCGTAAACCATCAAGAATGTCAG-3'
OsBC1L6-RNAi vector construction	RNAi_SpeI_*BC1L6*_F	5'-TAACTAGTTGTTCAAGAAGGACCCCAAG-3'
	RNAi_XbaI_*BC1L6*_R	5'-ATTCTAGATGTGTTTGCATCTTATCTTGTCTG-3'
genotyping	NF7793-F	5'-GGAGATAATCTGGGCGATGA-3'
	NF7793-R	5'-TGCACAAAAGAAATCAAGCG-3'
	Tos17-tail6	5'-AGGTTGCAAGTTAGTTAAGA-3'

*The underline indicates the added linker sequence.
