# Supplementary figures and images for: Oryza sativa Brittle Culm 1-like 6 modulates β-glucan levels in the endosperm cell wall
Source: PLoS One. 2019 May 23;14(5):e0217212. doi: 10.1371/journal.pone.0217212 (PMC6532911; doi:10.1371/journal.pone.0217212)

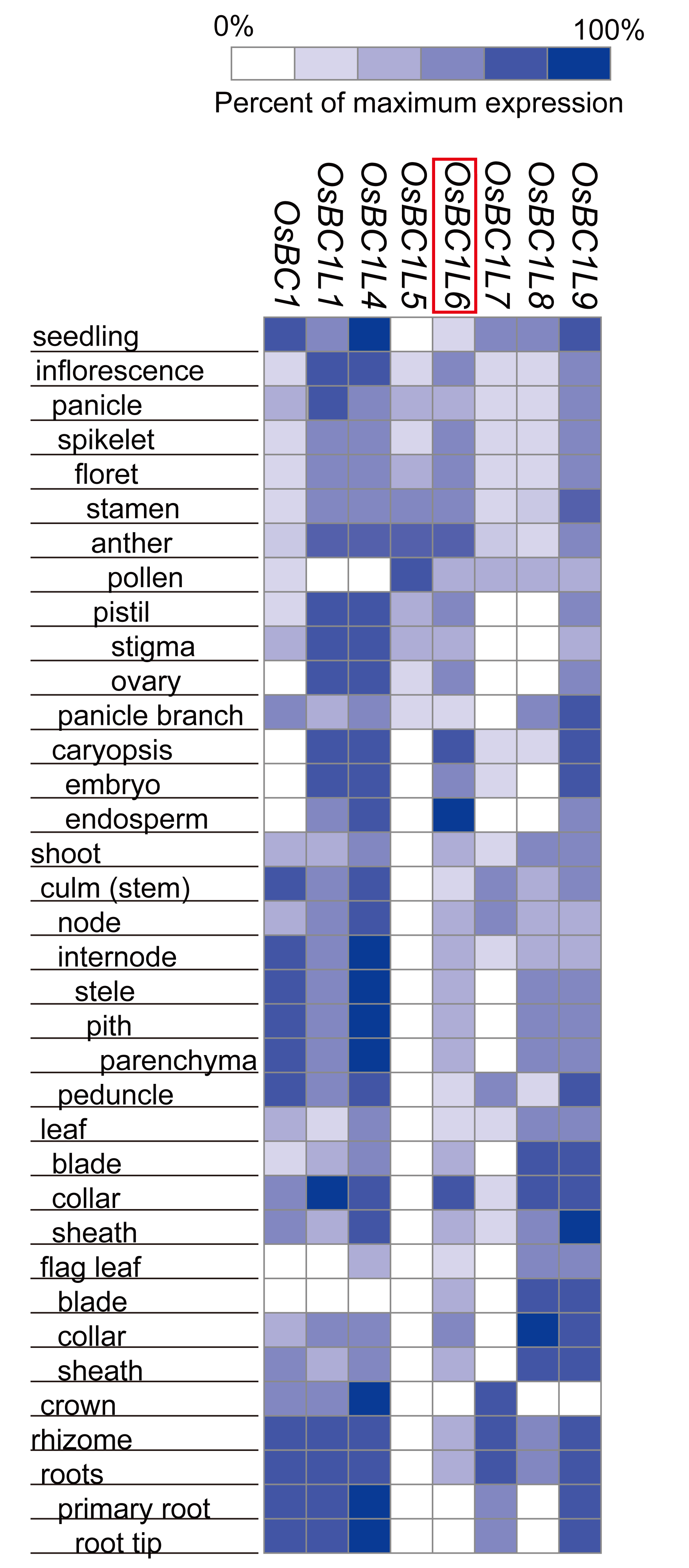

Supplement: S1 Fig — The expression patterns of OsBC1L family members in different tissues obtained from the DNA microarray database Genevestigator (https://genevestigator.com/). OsBC1L2, OsBC1L3, and OsBC1Lp1 are not listed because no probes for these genes are available in the GeneChip Rice Genome Array (Affymetrix). (TIF) [file pone.0217212.s003.tif]

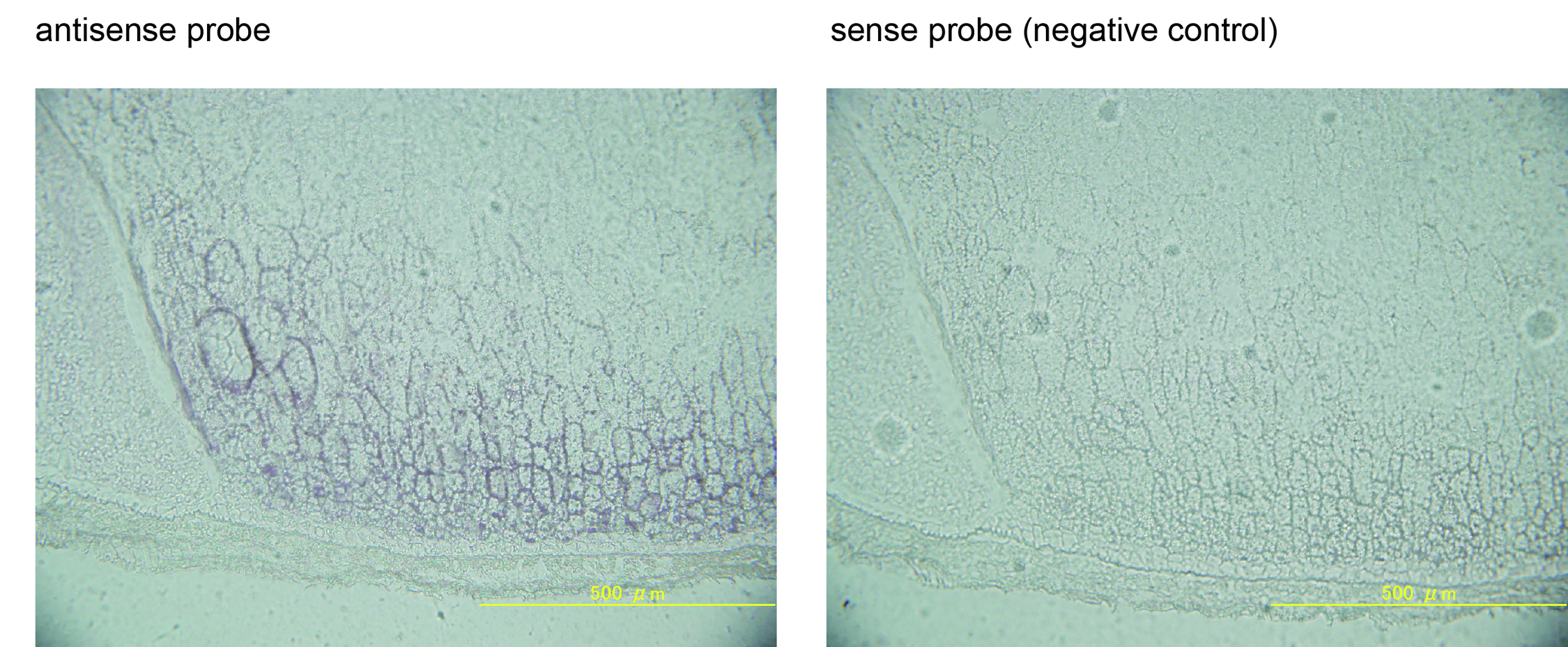

Supplement: S2 Fig — Sections of 15-DAF seeds hybridized with an OsBC1L6 antisense probe (left) and sense probe (right). (TIF) [file pone.0217212.s004.tif]

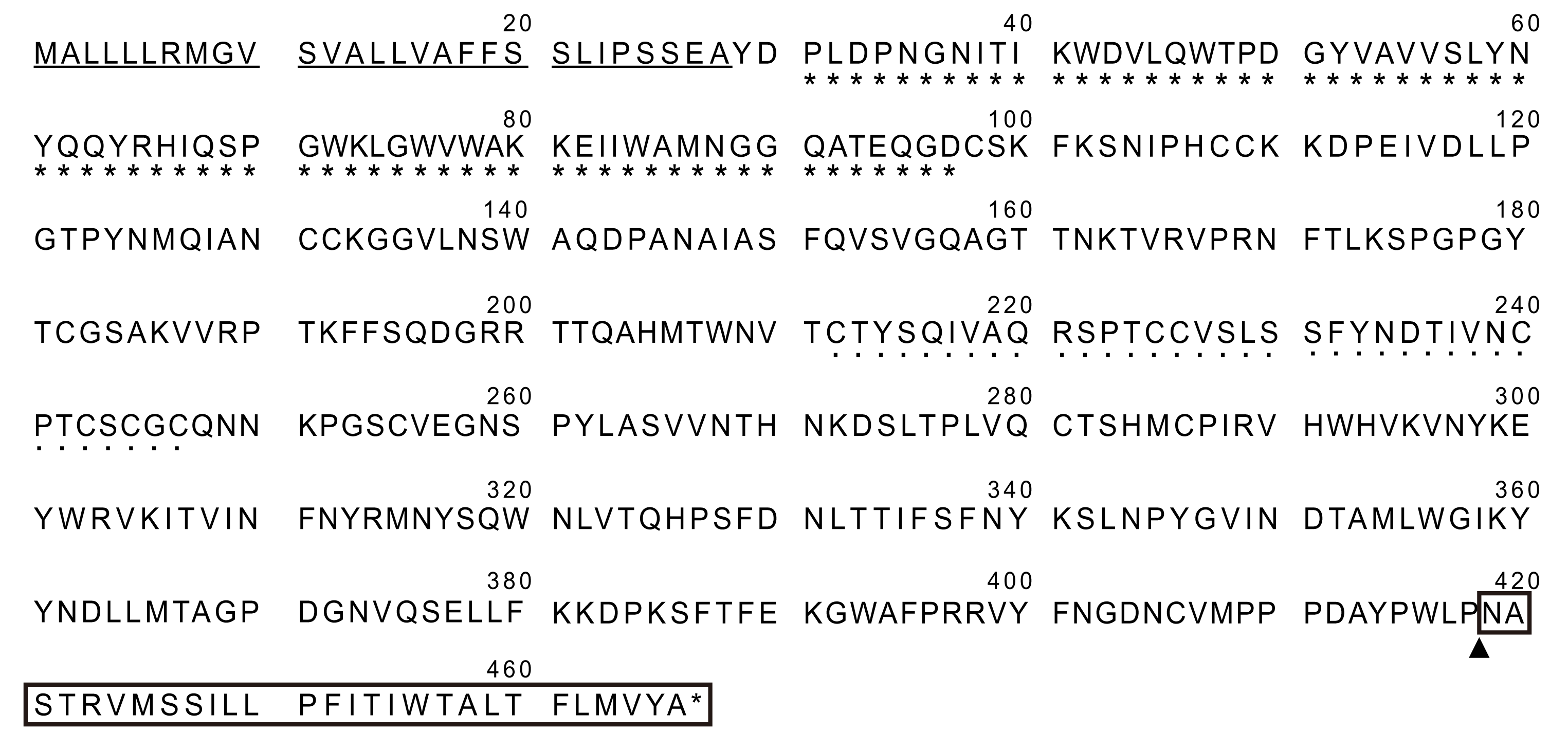

Supplement: S3 Fig — The predicted amino acid sequence of OsBC1L6. The signal peptide sequence is underlined. The region expected to be a polysaccharide-binding domain is indicated by asterisks (CBM). The dotted line represents the CCVS Cys-rich domain, which is highly conserved across the COBRA gene family. The GPI anchor domain is boxed. After translation, this domain is expected to be cleaved at the ω site (arrowhead) of the N terminus of the GPI anchor domain, and the GPI anchor is added. (TIF) [file pone.0217212.s005.tif]

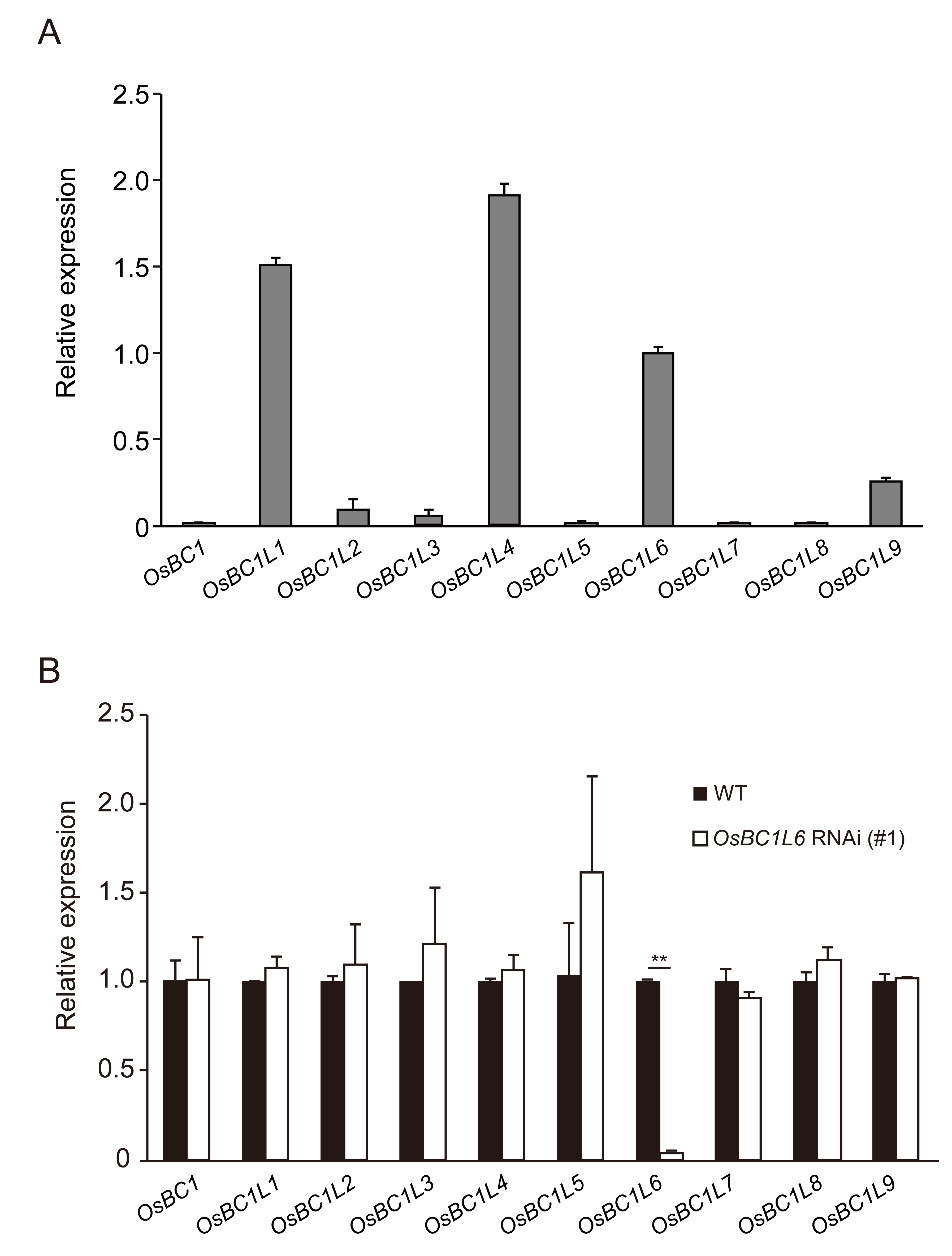

Supplement: S4 Fig — Total RNA was extracted from WT and OsBC1L6 RNAi calli (#1) and subjected to RT-PCR. (A) Expression levels of OsBC1L family members in WT calli. (B) In the OsBC1L6-RNAi line, the expression of OsBC1L6 was specifically suppressed. Values were normalized to the expression level of RUBQ and are shown as the mean ± SD. Asterisks indicate significant differences, as determined by Student’s t test (**p < 0.01, n = 3). The primers used in this experiment are shown in S1 Table. (TIF) [file pone.0217212.s006.tif]

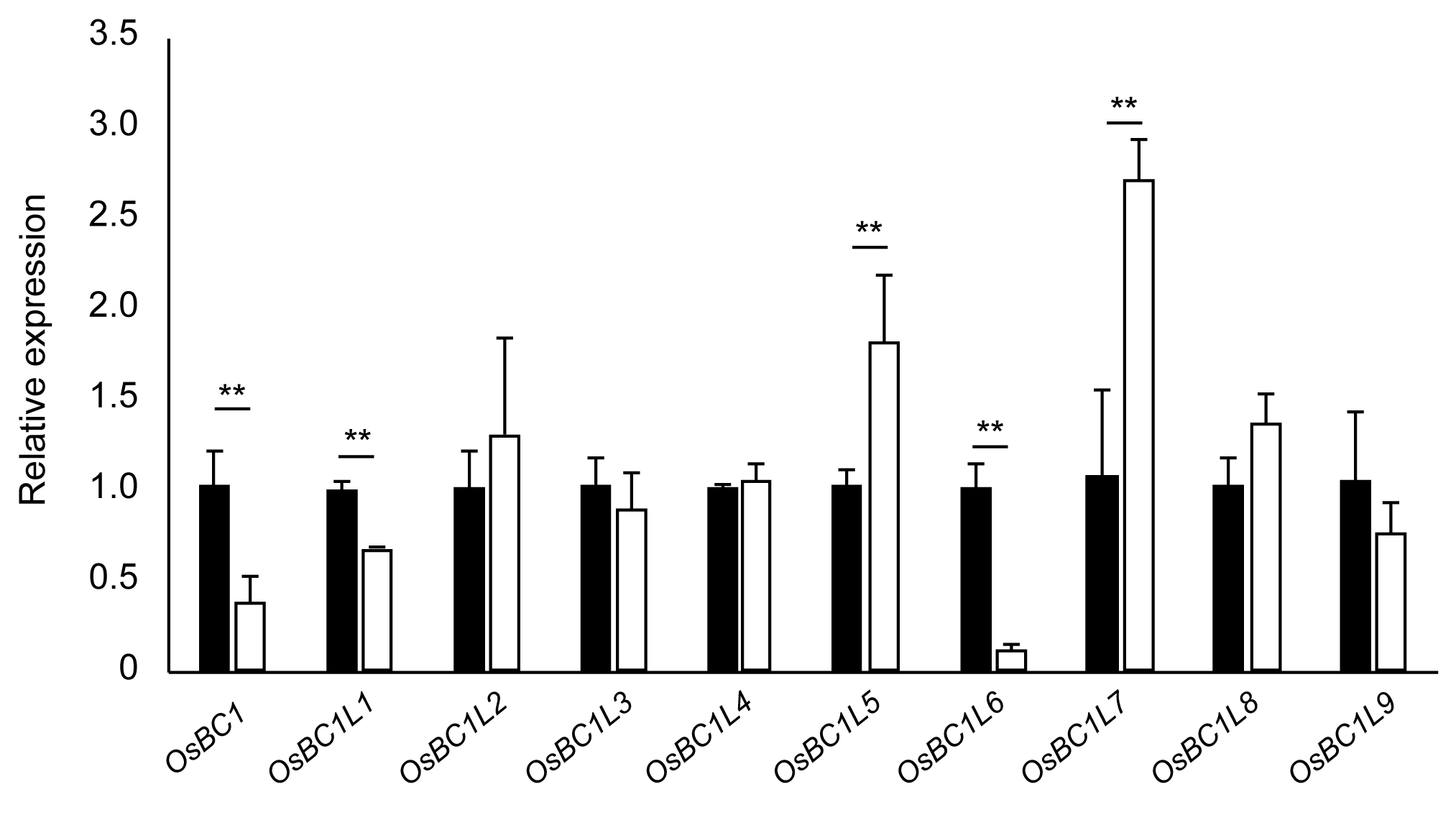

Supplement: S5 Fig — Total RNA was extracted from 15-DAF seeds of WT and the Tos17 insertion mutant (-/- 7–5) and subjected to RT-PCR. Values were normalized to the expression level of RUBQ and are shown as the mean ± SD. Asterisks indicate significant difference, as determined by Student’s t test (**p < 0.01, n = 3). The primers used in this experiment are shown in S1 Table. (TIF) [file pone.0217212.s007.tif]

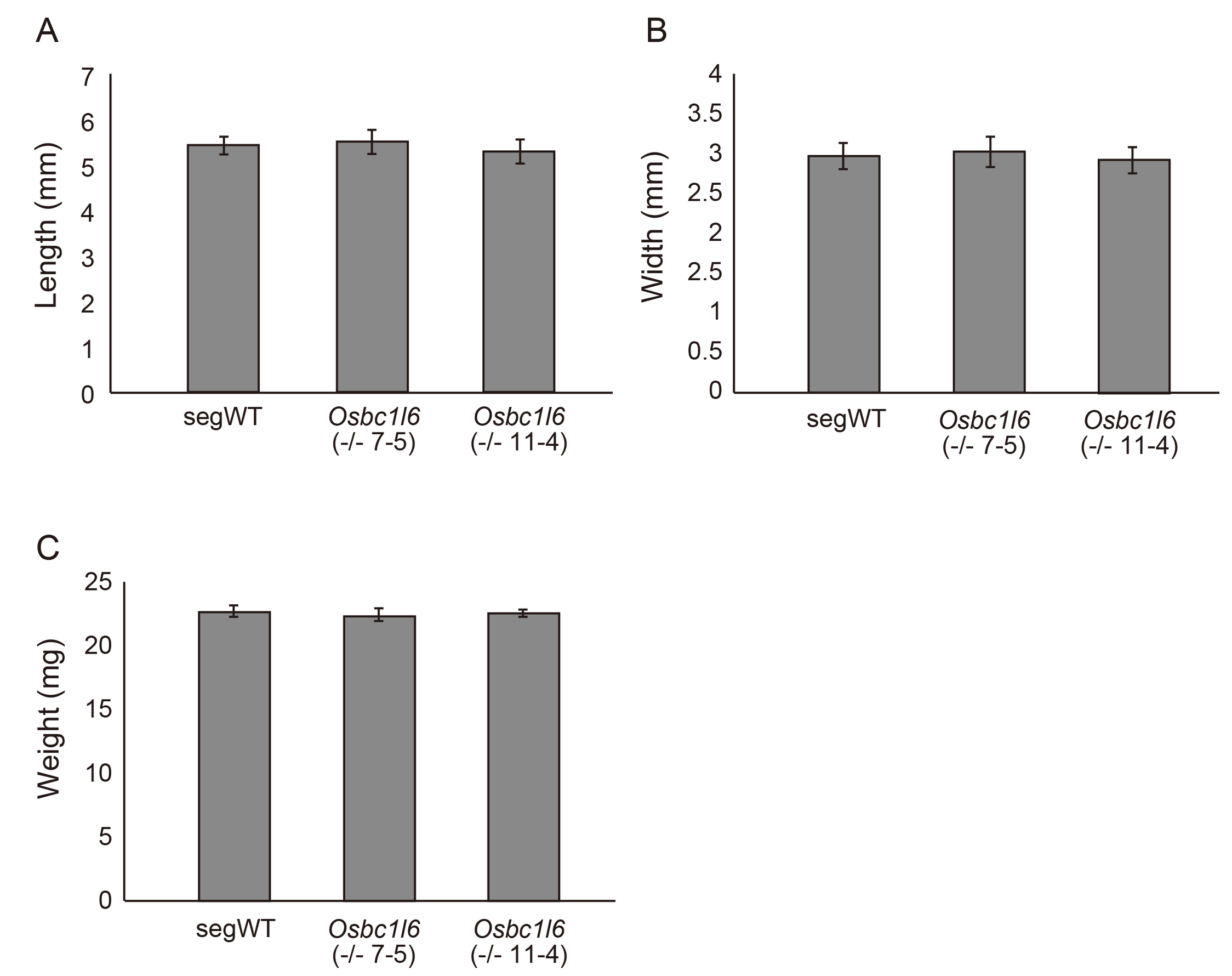

Supplement: S6 Fig — (A) Grain length. (B) Grain width. (C) Grain weight. Data represent the mean ± SD (n = 50). (TIF) [file pone.0217212.s008.tif]

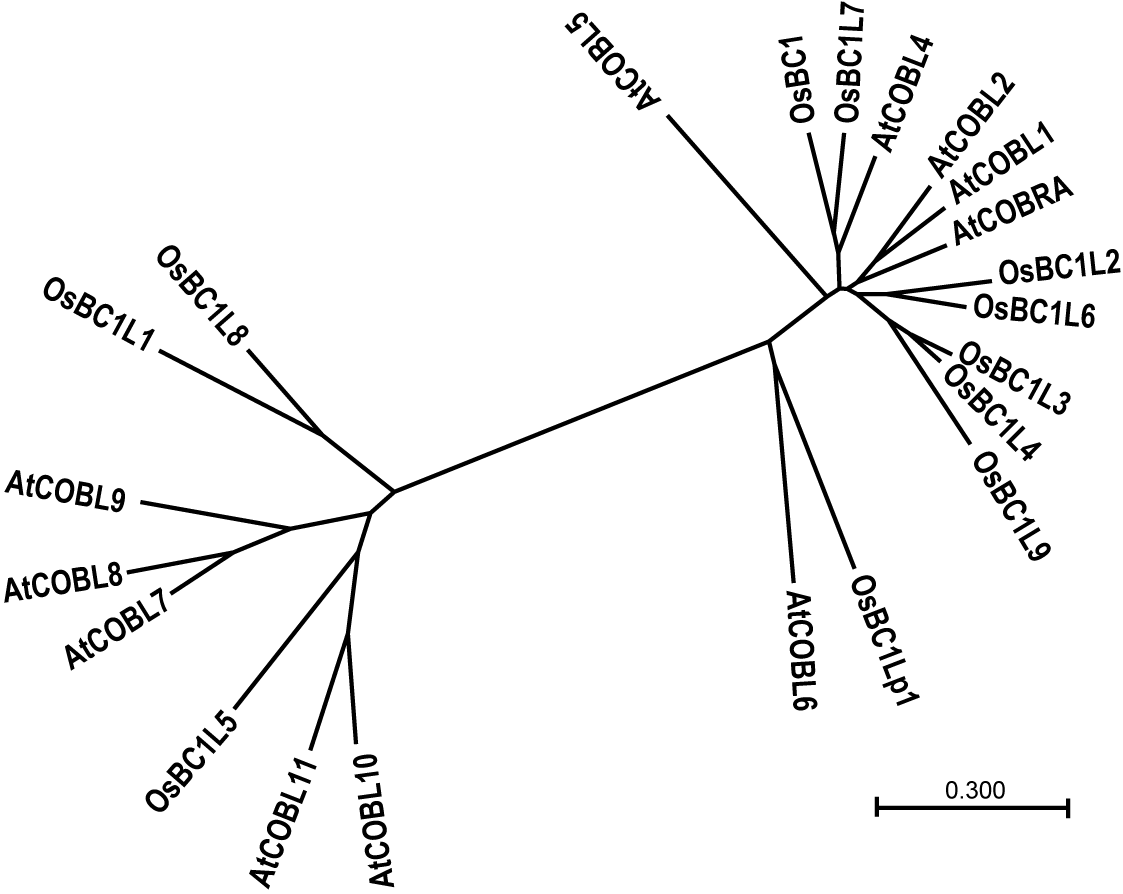

Supplement: S7 Fig — Phylogenetic tree based on the amino acid sequences of the COBRA family proteins constructed using the CLC Sequence Viewer (https://www.qiagenbioinformatics.com/products/clc-sequence-viewer/). The tree was created using the neighbor joining method. The protein sequences of AtCOBRA (Arabidopsis thaliana) and OsBC1 (Oryza sativa cv. japonica) family members were obtained from the NCBI (https://www.ncbi.nlm.nih.gov). The signal peptide was predicted using SignalP version 4.1 (http://www.cbs.dtu.dk/services/SignalP/) [20], and the hydrophobic profile was generated using TMHMM version 2.0 (http://www.cbs.dtu.dk/services/TMHMM/). GPI modification was predicted using big-PI Predictor [21]. GenBank protein ID numbers are as follows: Arabidopsis thaliana AtCOB, AAK56072; AtCOBL1, AAF02128; AtCOBL2, BAB02996; AtCOBL3, AAG12670; AtCOBL4, CAC01762; AtCOBL5, BAB10644; AtCOBL6, AAB60732; AtCOBL7, CAA74765; AtCOBL8, BAB00585; AtCOBL9, BAB10345; AtCOBL10, BAB01166; AtCOBL11, CAB38841; rice (Oryza sativa) OsBC1, BAS84702; OsBC1L1, BAS83769; OsBC1L2, BAS84703; OsBC1L3, BAS86439; OsBC1L4, BAS93810; OsBC1L5, BAS99187; OsBC1L6, BAT02549; OsBC1L7, BAT02550; OsBC1L8, BAT03332; OsBC1L9, BAT11495; OsBC1Lp1, BAS90288. (TIF) [file pone.0217212.s009.tif]
